# Development of an animal health testing tool to reduce antimicrobial use on farms: perceptions, implications, and needs of Irish dairy farmers and farm veterinarians

**DOI:** 10.1186/s13620-024-00268-x

**Published:** 2024-06-21

**Authors:** Karen McGrath, Áine Regan, Emer Kennedy, Tomás Russell

**Affiliations:** 1 UCD School of Agriculture & Food Science, University College Dublin, Belfield, Dublin 4, Ireland; 2 Department of Agri-food Business & Spatial Analysis, REDP, Teagasc Mellows Campus, Athenry, Co. Galway H65 R718, Ireland; 3 Teagasc, Animal & Grassland Research and Innovation Centre, Moorepark, Fermoy, Co. Cork P61 C997, Ireland

**Keywords:** AMR, Digital technology, Animal diagnostics, User-centred design, Design thinking, Behaviour change

## Abstract

**Background:**

The threat of antimicrobial resistance is triggering the need for behavioural change towards antimicrobial use on Irish farms. Newly introduced veterinary medicine regulations are mandating the restricted and more prudent use of antimicrobials in the animal health sector. The need to reduce antimicrobials has placed a greater emphasis on the importance of animal health testing, however, issues with current testing practices are affecting diagnosis and subsequent drug usage. There is potential for digital technologies to address these issues and reduce antimicrobial use on farms, however, for these tools to be successful, they would need to be developed in collaboration with future end users.

**Results:**

Using qualitative approaches (focus groups), this study engages with dairy farmers and farm veterinary practitioners to detail current challenges with animal health diagnosis and to explore the initial development of a rapid, on-farm animal health testing tool to address these challenges. Issues with timing and testing, the role of knowledge and experience, and veterinarian availability all affect the ability of farmers and veterinarians to diagnose animal health issues on farm. These issues are having negative implications including the increased and unnecessary use of antimicrobials. An on-farm testing tool would help mitigate these effects by allowing veterinarians to achieve rapid diagnosis, facilitating the timely and targeted treatment of animal illnesses, helping to reduce overall antimicrobial use on farms. However, engagement with end users has highlighted that if a tool like this is not developed correctly, it could have unintended negative consequences such as misdiagnosis, increased antimicrobial use, challenges to farmer-veterinarian relationships, and data misuse. This study outlines initial end user needs and requirements for a testing tool but suggests that in order to successfully design and develop this tool, co-design approaches such as Design Thinking should be applied; to mitigate future negative impacts, and to ensure a testing tool like this is designed specifically to address Irish dairy farmers and farm veterinarians’ values and needs, ensuring responsible and successful uptake and use.

**Conclusions:**

Digital tools can be effective in reducing antimicrobial use on farms, however, to be successful, these tools should be designed in a user centred way.

**Supplementary Information:**

The online version contains supplementary material available at 10.1186/s13620-024-00268-x.

## Background

Antimicrobial Resistance (AMR) is a globally recognised OneHealth issue which threatens the future of human and veterinary medical treatment. The development of AMR is caused and accelerated by an increased and overuse of antimicrobials in human and animal medicine [1]. Recognising AMR as a threat to public safety, the World Health Organisation (WHO) in 2012 set out a global action plan outlining strategic measures to slow AMR development, with a focus on the agricultural, animal health, and human health sectors [2]. In response to this action plan, the European Union (EU) implemented new veterinary medicine regulations [3] in January 2022, with the goal of reducing and regulating the use of antimicrobials on animal farms. The legislation mandates an end to the prophylactic use of antimicrobials, and sets out new rules for the supply, distribution, control and use of veterinary medicinal products and medicated feed, ensuring that antimicrobials can only be obtained on foot of a veterinary prescription [4]. Additionally, as part of the EU Green Deal, there is a commitment under the Farm to Fork Strategy, to reduce the overall sales of antimicrobials for farmed animals and in aquaculture by 50% by 2030 [5].

In light of these new restrictions, across the EU, the agricultural sector will face a number of challenges. Successful adherence to these new rules will necessitate a behavioural change and a shift in how farmers and veterinarians think about and use antimicrobials on farm. A ban on the prophylactic use of antimicrobials will require a more strategic use of vaccinations to prevent diseases and more timely diagnosis of animal illnesses to ensure appropriate antimicrobial use. It will also signal the need to switch from the routinely used practice of blanket dry cow therapy (BDCT) to more targeted selective dry cow therapy (SDCT) [1]. In Ireland, BDCT has long been used on farms and this practice has become culturally ingrained in farmers as a means to treat and protect animals from infections. Changing from BDCT to SDCT will present challenges to Irish dairy farmers, requiring a significant infrastructural, behavioural, and cultural change [1]. National efforts have been made to promote these changes, for example Animal Health Irelands CellCheck mastitis control program [6], and this has had a positive effect with sales trends indicating a reduction in the use of intramammary antimicrobial usage between 2015 and 2019 [7, 8]. However, despite this improvement, BDCT is still a very common practice in the Republic of Ireland [8]. Such data on antimicrobial use on farms will be needed to objectively monitor progress (McAloon et al., [8]) and whilst there are some sales data available on intramammary antimicrobial usage, there is no published data available to estimate the overall quantities of antimicrobials used in the Irish dairy industry [7]; which under new EU regulations, will become a requirement. This lack of available data is a challenge that will need to be overcome in order to better quantify and manage antimicrobial use within the Irish dairy sector. However, the practice of collecting robust and accurate data in the sector will bring its own set of challenges, such as veterinarian and farmer motivation to monitor animal health and antimicrobial usage [7].

As the EU regulations come into force, Irish veterinarians are under increasing pressure to prescribe antimicrobials more prudently and responsibly [4] meaning measures to support high standards in antimicrobial stewardship will be needed [9]. These implications will mean that maintaining good animal health status in Irish herds and controlling and preventing disease outbreaks on farms will become paramount. These parameters can be achieved via early diagnosis of animal health issues, signalling the need for more adequate animal health testing practices on Irish farms.

### Current animal health testing practices

Some of the main animal health issues on Irish dairy farms include mastitis, lameness, and calf health issues (diarrhoea and pneumonia) [10]. Whilst these health issues can frequently be detected by the naked eye, in most cases, their causative agent requires identification via diagnostic testing. A number of on-farm and in-lab tests are currently available to farmers^1^ and veterinarians^2^ respectively, but for other diseases, normal practice requires samples being sent to Regional Veterinary Labs (RVLs) for analysis. Conducting on-farm tests and submitting samples to RVLs will be important to monitor animal health issues on farms and to prevent disease outbreak.

However, there are limitations on the range of on-farm tests available, and as farmers and veterinarians determine which cases to submit to RVLs for testing, there is a risk that under-reporting of cases and lack of engagement with testing procedures by farmers and veterinarians could impact disease prevalence on farm. Research has explored factors which affect farmer and veterinarian engagement with animal health testing, some of which include type of case, influence of the veterinarian, inconvenient RVL opening hours [11], distance of farm to the RVL, and past issues with RVL services including receiving inconclusive results, long wait times in receiving test results, or not receiving test results at all [10]. Farrell and co authors [12] have found that delays in test results can increase antimicrobial use, therefore highlighting a need for more rapid testing methods in the industry.

### What role might technology offer to alleviate some of these challenges?

Digital technologies have the potential to speed up animal health testing. Developing a digital tool that could enable rapid on-farm animal health testing would allow veterinarians to achieve early diagnosis and tailor the treatment of that animal accordingly. A diagnostic tool like this would likely catalyse significant behaviour change on farm and help farmers and veterinarians to reduce antimicrobial use, slowing AMR [12]. This type of tool could promote farmer engagement with sample testing by making the process more efficient, and the ability of veterinarians to use a rapid testing tool on farms would resolve barriers such as distance to RVLs and wait times for results coming back from the labs.

Technology developers are looking at the potential of precision livestock farming (PLF) for more efficient and targeted animal health testing, however, for these tools to be successful, they will need to be developed in a user-centred way. The majority of PLF innovations are designed and developed from a technical and scientific point of view, with little or no input from the end user [13]. Research has found that these ‘top-down’ approaches can negatively affect successful technology design and end user adoption as technologies are not designed to specifically address user values and needs; factors which should be the drivers of technology research, design, and development [14]. Recent research has called for more inclusive approaches to technology design and for research to engage designers and engineers with end users during the design process [15, 16]. Using these approaches will ensure that technologies are user centred and that the needs and values of stakeholders will be considered and built into the design of these tools; all of which should promote successful user uptake [17].

### Current study

The current study employs a user centred design approach to explore the potential development of a rapid on-farm animal health testing tool. The concept of this diagnostic tool is being developed in Ireland in a collaborative project between VistaMilk Research Centre^3^ and Tyndall National Institute.^4^ It is anticipated the tool will be a handheld device which uses biological sensors to perform on the spot sample testing which traditionally would need to take place in an RVL. These sensors will be able to determine whether a sample is positive or negative for an animal illness or disease, through its ability to identify and detect antibodies in that sample. The idea for this tool is that the desired sensor microchip (which correlates to what you wish to test for) is inserted into the device, and the sample you wish to test (e.g., blood/milk/mucus) is also placed into the device for testing. The device will be linked to an app on the users phone which will be used to ‘tell’ the device to start testing. When the device is finished testing the sample (approx. 10–15 minutes), results for that sample are sent back to the app, indicating to the user whether the sample is positive or negative for a given disease/illness. This rapid detection would allow veterinary personnel to identify illnesses early and to determine the correct treatment plan for that animal.

The successful development and integration of this tool could have positive effects for the Irish agricultural industry as it eliminates wait times for blood results returning from labs and assists veterinarians in diagnosing and treating sick animals quickly and correctly at the time of call-out. It would aid veterinarians in determining the correct (if any) antimicrobials to be administered, eliminating unnecessary use of antimicrobials and safeguarding our critical antimicrobials to only be used when absolutely necessary. This would tackle antimicrobial resistance issues and improve animal health and welfare standards on Irish farms.

This study has two key aims. Firstly, it will examine Irish farmers and veterinarians’ attitudes towards the current process of diagnosing animal health issues on farms, paying particular attention to barriers to diagnosis. It will then explore the potential of, and farmer and veterinarians’ perceptions of, a digital testing tool to address these barriers.

## Methods

### Sample selection

Data were collected through focus groups (*n = 4*) conducted in the South and Southeast of Ireland between January – April 2023. A total of 33 participants consisting of 31 males and 2 females took part in the study. Of the four focus groups, three were comprised of dairy farmers (*n = 23*) and agricultural advisors (*n = 2*), and the fourth of farm animal veterinarians (*n = 8*). Purposive and convenience sampling techniques were used to recruit the target population of dairy farmers and farm animal veterinarians. No other parameters for participation were used in an effort to have groups as naturally diverse and open as possible. Agricultural advisors were used to recruit for two of the three dairy farmer focus groups, with the third being conveniently sampled through the first authors personal network. The veterinarian focus group was recruited via a network of independently owned veterinary practices on the island of Ireland. Potential participants were sent information regarding details of the wider study and specific objectives of the focus group, and if expressions of interest for voluntary participation were received, a time and location was set for each group.

### Data collection procedure

A structured interview schedule was developed by the research team to guide the focus group discussion. Similar to the approaches taken by Kenny and Regan, [13] and Hammersley and co authors [18], the interview schedule began with introductory questions and an interactive activity to introduce participants to the topic of discussion and get them comfortable with it. This was followed by key exploration questions to get to the heart of the discussion, and concluded with closing questions that sought any further comments regarding the topic, and to check if anything was missed.

A focus group interview schedule was drafted by the first author and refined based on discussions with the wider research team. This interview schedule was then pilot tested with a group of agricultural research students and technical staff (*n = 7*) on University College Dublin’s research farm in December 2022. Following participant feedback, analysis of collected data, and consultation with the research team and industry personnel, interview questions were refined and/or rephrased for better understanding and to better address the key objectives of the study. The interview schedule underwent two rounds of modifications and refinements, and the finalised version can be found in Appendix A. Focus group questions were developed to address the aims of the study which were to 1) explore farmers and veterinarians’ attitudes towards diagnosing and detecting animal health issues on farms, and 2) to explore farmers and veterinarians’ perceptions of the use of digital tools to address these concerns, pitching the concept of a digital testing tool as basis for discussion. The same interview questions were used for both the farmer and veterinarian focus groups, however, small refinements were made to how they were phrased to relate to each groups contexts.

All focus groups were conducted by the same researcher to ensure uniformity and transparency in the interviewing style. Each focus group took place in either a place of work (veterinary practice or on-farm), or in a local community setting as agreed with each group. Focus group size varied between 5 and 11 participants and lasted between 25 and 80 minutes. Focus groups were conducted until theoretical saturation was reached; whereby no new or relevant data was identified [19]. Data were collected verbally through group discussion but also in material form, using sticky notes, markers, and flipchart sheets. Each focus group was audio recorded using a digital recorder.

### Analysis

Focus group recordings were manually transcribed verbatim and anonymized by the first author. Field notes such as noting pauses and the tone of certain remarks were added to each transcription to help contextualise verbal accounts, and to gain a more nuanced understanding of each discussion. Each of the transcripts were imported to NVivo 12 and thematically analysed using an inductive thematic approach [20]. Firstly, the transcripts were read and re-read by the first author to allow them to become fully immersed in the dataset. During this step, additional notes were taken on any observations made. Next, initial codes were developed and recorded, forming raw data for analysis. These codes were reviewed and organised into broader themes and subthemes, which were then further reviewed, refined, and defined. The themes were then reflected upon to ensure that they were representative of the data, after which the most compelling quotes that accurately represented experiences of the participants were noted to represent each theme.

## Results

**Fig. 1:**
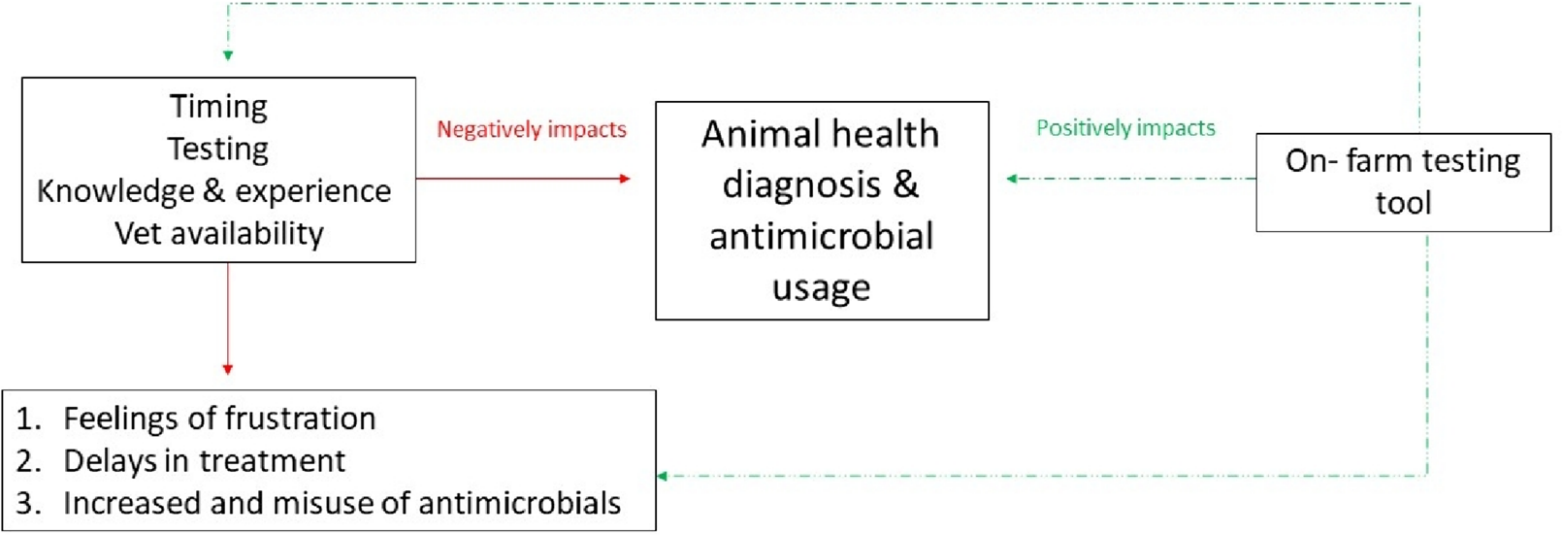
Thematic Map

Section one outlines farmers and veterinarians’ perceptions of the key animal health issues on Irish dairy farms, as well as key barriers to their diagnosis. Section two describes farmers and veterinarians’ perceptions of the potential of a digital tool to aid with on-farm diagnosis of animal health issues Fig. 1.

## Section 1: Farmer and veterinarians’ perceptions of current issues with diagnosing animal health issues on Irish dairy farms

### Current attitudes towards animal health diagnosis

Respondents in this study found that timing, testing, knowledge and experience, and veterinarian availability are key issues affecting animal disease diagnosis on Irish dairy farms. Respondents indicate that these barriers can have some negative impacts including feelings of frustration, delays in animal treatment, and increased use or misuse of antimicrobials on dairy farms. The following section describes these issues in more detail and outlines the implications that these issues are having.

#### Issues of timing and testing

Both farmers and veterinarians indicate that the most important factor involved with diagnosis of animal health issues is time. Participants express the need to be able to identify and treat an illness *as* it is developing in an animal. Some precision livestock technologies such as cow collars and automatic calf feeders alert farmers to a problem at an earlier stage of disease progression allowing for earlier action and treatment. However, farmers who do not use these tools are left “*waiting for the tell-tale signs”* but by then it is *"too late"*; when one animal is down, they expect more to follow.

Farmer 7: *“well sometimes it might happen [that you have to] let a thing develop to find out what it is”.*

The use of monitoring tools also proves valuable for veterinarians, resulting in earlier and improved quality of call-out, giving them a better chance of successfully treating the animal. However, whilst veterinarians praise the effectiveness of monitoring technologies in animal health detection, they critique the absence of appropriate animal health diagnostic technology.

Veterinarian 1: *“so the collars are doing their job, as in traditionally we would see those cows 24, 36, 48 hours later, so the collars are doing their job very well, they work extremely well. Our frustration is the cow side testing afterwards, that we can’t get results quickly enough. By the time we get the results, that cow is dead and there’s probably 10 more sick you know. That’s the frustration really.*

Veterinarian 3: *but still for [farmer name] that [monitoring] technology is of benefit to him (…).*

Veterinarian 2: *oh ya his cows are surviving because of the [monitoring] technology, the problem is we can’t stop what’s happening because [the] testing technology is not appropriate”.*

Problems with testing are raised by both farmers and veterinarians and issues include delays in getting test results back, or results coming back inconclusive or providing no insightful answer.

Agricultural Advisor: *“probably the most frustrating I hear from the lads is if a calf dies or say for some reason, [sent samples] into the regional veterinary lab, and then weeks later, inconclusive or something like that”.*

Veterinarians especially convey a deep frustration and exacerbation with testing delays. Both farmers and veterinarians describe that these possible delays could be down to logistical issues with the RVL, samples going missing, or the RVL acting as a contractor instead of conducting the testing themselves.

Veterinarian 2: *“the issue is, so I took swabs right, and swabs went to [location] RVL. Subsequently I had discovered that the swabs then had to go to two different labs after that – one for virology, one for bacteriology. That’s not stated anywhere”.*

#### Role of information, knowledge, and experience

Farmer knowledge and experience can act as a facilitator and barrier to disease detection and diagnosis on farms. Prior experience of having a particular disease on farm would enable farmers to be familiar with its clinical signs and therefore better equipped to recognise and treat it if it reoccurred. However, the same can be said for the inverse of this statement; lack of prior experience of an illness will limit farmers ability to detect and diagnose it.

Farmer 3: *“The more of either or any of them you get I suppose the more savvy you become with it let’s say so if someone has issue with lameness a lot, you kind of nearly know to look at a cow what it is, you know you, you just become more familiar with that particular (pause) whereas there’s certain [diseases] there thankfully I haven’t got and I wouldn’t know a whole lot about it until I’m affected by it”.*

Awareness also falls into this bracket as farmers may not even be aware that a particular disease exists. This was the case for one farmer who had an outbreak of mycoplasma mastitis. At the time, the farmer thought that his animals had very bad *e-coli* mastitis but could not work out why his animals were not responding to antimicrobials and were subsequently dying. The farmer spoke of his struggle to identify what it was as it was an illness that he had no prior knowledge of.

Farmer 2: *“Well, I suppose awareness was the problem there because I didn’t know anything about it. T’was a new one to us, we didn’t have any knowledge of it really.”*

This lack of knowledge and experience can be thought about in a number of ways. Firstly, it can be viewed as a positive as a farmer would not have had an outbreak of that disease before. Some view it and speak about it in a more matter of fact, self-explanatory way self-professing *“we’re not equipped for that”, “we’re not vets like”.* However, others are more self-reflective in this thought and highlight that this lack of knowledge regarding animal health is something that needs to be improved on and that technology can help to bridge that knowledge gap.

Farmer 4: “*I think it’s our own experience too, we need to gain that experience. We’re lacking there, maybe it’s just the education we’re lacking in, I don’t know. We’re looking for the technology to bring us that knowledge”.*

#### Lack of and misuse of information

A key barrier for veterinarians in diagnosing illnesses is the lack of information they receive at the time of callout. Veterinarians explain that when they arrive on farm they try to get as much information about the animal as possible as a lot of the diagnosis will be based on that information. On farms that use monitoring technologies this information can be available, however, veterinarians say that often times they arrive on farms and receive limited or no history on the animal, impeding their ability to diagnose.

Veterinarian 4*: "the collar [works] very well in that form and sense of things because when you get frustrated normally when you walk out to a farm and it's either [the farmer’s] not actually milking the cows anymore, so he just knows the cow is off and you ask is she eating? ‘I don’t know’ (farmer response). Did she milk this morning? ‘I don’t know’. Did she milk yesterday? ‘I don’t know.’*

Veterinarian 1: *or he hasn’t seen her for 3 days ya.*

Veterinarian 4: *(…) it’s usually a breakdown of the history behind the cow that’s when we get most frustrated is what I think anyway. But like when you ask the man there or it might be the fella working and doing the tractor work and there’s a cow in the crush and he hasn’t a clue he’s just [pointing to indicate being shown] ‘that’s the cow’.*

Veterinarian 1: *or there might be no one there, you might be told where the cow is and work away”.*

Veterinarians also indicate that although some farmers have adopted and invested in monitoring technologies and have access to detailed information, it does not always mean that they are utilised properly.

Veterinarian 1: *“obviously the equipment is only as good as the user. I saw two cows for a fella on Sunday morning and they were definitely sick for several days and they had collars on them and I said, ‘did you not get an alert?’ and he says, ‘you see I don’t look at that often enough’, so the technology is only as good as the user”.*

#### Veterinarian shortages

Farmers acknowledged the current shortage of farm animal veterinary practitioners in the country and indicated that fewer veterinarians and larger herd sizes mean veterinarians are busier now than they used to be. Farmers discuss the impacts this is having including the unavailability of veterinarians to call to the farm as often as they would like. One farmer says that it has reached the point where a veterinarian in a nearby locality is asking clients to send on videos and pictures of what is wrong with the animal and *“nearly diagnosing by FaceTime”* because he is so busy and cannot get to the farm. Delays in veterinarians arriving at farms will have impacts on animal health and ability to get early diagnosis. These examples give an insight into the real-life impact this shortage is having and how it manifests in practice. This highlights a real area of concern for the future of veterinary practitioners and their capacity to work on Irish farms.

### Knock on effects of late diagnosis

These issues with diagnosis are having negative knock-on effects for farmers and veterinarians. These include feelings of frustration, delays in treatment, and increased/misuse of antimicrobials.

#### Frustrations and delays

The main feelings farmers and veterinarians expressed when discussing diagnosis were frustration. The source of these frustrations came mainly from the delays in testing and the implications these have for farmers and veterinarians.

A delay in testing will delay diagnosis, which delays treatment. Veterinarians say they are reluctant to do anything that will cost the farmer a lot of money until they have hard data to say that there is definitely something wrong with the animal, leading veterinarians to *“firefight”* illnesses on farms until they get a conclusive answer.

Veterinarian 2: “*Well, we’ve this guy, he’s a great farmer, and everything is done right (…) and we’ve had this massive outbreak of a mystery, presumed virus. You know we’re sending off samples and stuff, there’s a huge time delay in getting those back. And we still haven’t found out a month later what said virus is so we’re just firefighting (…)”.*

These delays in treatment have negative knock-on effects such as deterioration of animal health, spreading of disease to other animals, and animal mortality. The extent of delays in treatment is highlighted by this farmer; *“it can take a long time to get blood results back. We had a bull last year, took about a week to get them back and he died in the meantime like”.*

#### Increased/misuse of antimicrobials

Reliability and variability of testing is a highlighted issue with farmers explaining that they could get different veterinarians from the same practice giving two different diagnoses. Veterinarians also experience this inconsistency of testing explaining that previously they had sent the same sample to two different labs to see whether they would come back with the same results and they did not. This variability in results can lead to misdiagnosis which in turn can lead to an increased or misuse of antimicrobials on farms.

Advisor: *“wrong diagnosis as well like you know. By the time you have the information it could actually be… you know, as [Farmer 1] was saying in terms of mastitis, you could be treating it for the wrong mastitis, it’s too little too late you know by the time you get the information”.*

Delays in results can also increase antimicrobial use as Farrell and co authors [12] find, in their study on Irish veterinarians’ behaviour to antimicrobial use on Irish dairy farms, that as although veterinarians do not have the results, they will more than likely administer antimicrobials anyway. This is echoed by veterinarians in this study that say that when collars indicate a potential issue with an animal, “*the toughest call is to say there’s nothing wrong with them (2 other veterinarians agree), it’s a lot easier going and getting an injection and giving something”.* These viewpoints are supported by O’Connor and co authors [21] who find that Irish veterinarians would be willing to prescribe antimicrobials prophylactically if they thought that it would prevent disease.

### Key needs regarding diagnosing animal health issues

Farmers and veterinarians have the same goal for diagnosis; to achieve *early* diagnosis. Participants express the same key need; to quickly find out what the illness is and what exactly is causing it, so that they can accurately and quickly treat it. Veterinarians especially stress that anything that could speed up diagnosis would be a huge advantage on farms.

## Section 2: Implications of a diagnostic tool

During the focus groups, the concept of an on-farm diagnostic tool was pitched to and discussed by both farmers and veterinarians. The following themes outline farmers and veterinarians’ perceptions and views on the tool, and some of the implications (both positive and negative) this tool would have in practice both for animal health testing and animal health and welfare more broadly.

### Perceived value of a digital diagnostic tool

#### Initial reaction and identified benefits

Both farmers and veterinarians reaction to the concept of this tool were positive overall, with phrases such as *“invaluable”* and *“huge advantage”* being used by participants. Veterinarians especially had an overwhelmingly positive reaction to the tool and saw it as something that would be hugely useful to them. They expressed an intrigue that a tool like this would actually work, but that it would be a “*game-changer”* and a “*no-brainer*” if it did. Farmers also expressed similar sentiments, and both identified that the key value of this tool is that it would speed up diagnosis on farms; a key issue previously identified by participants. Participants also identified that the tool would be valuable if it could determine different strains of an illness. This would facilitate timely and targeted treatment, helping to reduce unnecessary antimicrobial use and ensure that critical antimicrobials are only used when necessary. This would thereby satisfy participants key needs relating to animal health diagnosis.

When considering more specifically where this could be used on farm, participants would see huge value if it could help them determine the particular strain of mastitis and calf scour, with veterinarians especially seeing it as being hugely significant if it could determine different strains of respiratory illnesses on farms.

Farmer 3: “*I definitely think (…) mastitis like t’would be, you could have your two different types of antibiotic tubes and you know which treatment to go in with straight away like just from a milk sample say compared to maybe waiting a week for the (pause), and then being…*

Farmer 5: *wrong on it”.*

Veterinarian 1: *“but if it did respiratory alone (veterinarian 2 agrees) it would sell. Well just to even rule out that it’s not RSV, that it’s not IBR, or it’s not BVD (veterinarian 2 agrees).*

Veterinarian 7: *or it is and you can go in and get the (…) vaccine straight away”.*

Additional benefits identified by farmers were that it could help them to get antimicrobials without needing to call out the veterinarian, it would bring all on-farm tests already available into one format, it would avoid issues with samples going missing, and it could help to reduce veterinarian workload. Another farmer identified that a tool like this would tie in with SDCT.

#### Utility

However, for some farmers, their positive reaction was coupled with an air of caution and scepticism, with some farmers critiquing its ability to do what it promised; *“must be an awfully great machine to test for everything with all the different [sensors]*” (said with scepticism). Whilst seeing its merit, other farmers questioned whether they would get full utility of the tool. As farmers are not licensed to take blood samples, some saw it as being more of a veterinarians tool, adding that a tool like this is “*not preventative, it’s reactive”,* and that *“it’s probably better for the farmer to have the monitoring tool and the vet then to have the matter of fact”.* Echoing this, another farmer said that “*if you’re going to use [the device] I’m sure you’d be hoping it’ll tell you that the animal has it before you can see it physically in the animal, couldn’t see much point in it if it didn’t do that like”.*

One farmer also mentions that the average farmer is only going to think of a handful of illnesses to test for and so may not achieve the full potential use of the diagnostic device. Veterinarians indicate that they would be surprised if this device was something that farmers would go for because they think that generally farmers are not into testing things, referencing a past initiative that was brought in for on farm culturing of milk samples which had minimal uptake. However, they imagine that farmers would love it if they (veterinarians) had it. Veterinarians go on to say that if this device is something that a farmer would get regular use of, then there are bigger herd health issues that need to be addressed on farm. Some farmers question the need of having this device at all, saying that experienced farmers and veterinarians will know what an illness is to look at an animal.

Farmer 5: *“you go to an older vet that has been in the game 40 or 50 years like, sure they’ll know looking in the shed, from their own experience they’ll know exactly what it is so they’ll think I don’t need a machine to tell me that like”.*

### Potential concerns/barriers

Despite the positive intention of, reaction to, and identified benefits of the technology, farmers and veterinarians expressed concerns in relation to its use and identified areas where this type of diagnostic tool could have unintended negative consequences if its use is not safeguarded. These negative consequences include misuse and overuse of antimicrobials, potential challenges to farmer-veterinarian relationships, and concerns over the use of data.

#### Misdiagnosis and risk of overprescribing

One of the main appeals of the device is that it could lead to more targeted and reduced use of antimicrobials on farms. However, some farmers and veterinarians identified the possibility that this device could potentially have the opposite effect, and result in an increased or misuse of antimicrobials. Speaking about the reliability of testing, both farmers and veterinarians expressed concerns that if this device does not work correctly, there is a risk that it will give a misdiagnosis which will negatively impact antimicrobial use.

Farmer 5: *“If the accuracy of it is not right it could destroy ya”.*

Other concerns arise from a focused discussion on farmers use of the technology. Veterinarians explain that an incorrect sampling technique, which could be implemented by inexperienced farmers, could lead to contamination of the sample, and could negatively influence test results and treatment. In the following quote, one veterinarian explains the consequences of how this happens already in practice.

Veterinarian 1: “*so in general, when any of our farmers, when most of our farmers take milk samples and we culture them, we generally culture what’s on the outside of the teat as opposed to what’s in the milk because they don’t do it cleanly. (…) they just get e-coli from the outside so that’s very frustrating. So equally if they don’t do it properly, we’re going to get erroneous results, and then you start prescribing stuff that’s just plain wrong, that would be unfortunate”.*

One group of farmers identified that instead of helping them to reduce antimicrobial use on farms, the presence of this device could lead to farmers overusing antimicrobials, as one positive result from the test could lead them to seek out antimicrobials and blanket use them as a prevention tool.

Farmer 2: *“I don’t think that will help on a farm to limit antibiotic use because you’ll say, ‘yea I’m after diagnosing a calf here, give me enough stuff for 30’ you know what I mean.*

Farmer 1: *and I’ll do the rest of them just in case.*

Farmer 2: *ya you will like because you don’t want the rest of them to get it (someone agrees) so you’re going to go in there and you might think they have it and they haven’t it so I’d say it will probably be, to avoid concerns, t’would be one of them anyway because you’d be over prescribed drugs like”.*

Building on this point, they later go on to say that a tool like this could be a risk because you are self-diagnosing. However, farmers then go on to say that they do not think that they will ever be given that kind of control over antimicrobials.

#### Challenges to farmer-veterinarian relationship

Farmers say that this tool could act as a stopgap to veterinarian shortages in the country but question the impact that it could have on farmer-veterinarian relationships. In their discussions, farmers demonstrated a consideration of and interest in what veterinarians would think about this tool. Farmers queried whether veterinarians would view the tool as a threat or as something that might be positioned, in a way, to replace them. They also questioned whether veterinarians would feel that they are being "*skipped over"* if farmers used this device in their stead and only contacted them to get antimicrobials. However, the general accepted consensus was that even if farmers had the device, the veterinarian is still needed and involved. Veterinarians have a better understanding of the limitations of diagnostic tests and so farmers recognise that even if they (the farmer) had primary use of such a diagnostic tool, they would require support from their veterinarian to correctly interpret test results and advice on what to do next.

Farmer 11: *“I’d say the vets are going to hate it because if it does work right then we won’t need them at all”.*

Farmer 9: *“What’s the vets thinking? Is it taking them out of work?”*

One group of farmers questioned whether a device like this might cause a strain or a tension between the farmer and the veterinarian if there is a discrepancy between what the veterinarian thinks the illness is and what the device says it is. This might call into question what the veterinarian will treat or at a more extreme level, the competency of the veterinarian. Some farmers initially agreed that it could be a problem and potentially damaging to a relationship that has been built up over years, however, farmers overall concede that the veterinarian will make the right call, and that in cases such as these, oftentimes, there is no such thing as a definitive answer. Veterinarians also agreed that they are not always right and are open to being wrong but that they could not imagine there being much difference in what they suspect from their clinical examination and what the device confirms.

#### Data use/misuse

Concerns regarding data use and data sharing surfaced during discussions. Veterinarians more so than farmers queried what happens with the data that is collected via the device, assuming that the gathered information will be sold off by the manufacturer; used to examine if veterinarians were prescribing antimicrobials responsibly and to produce national trends of antimicrobial use on farms.

Veterinarian 3: *“I imagine a significant source of the income for these people is selling this information at the end of it, this is how they justify having it, everything is for sale now, any information is for sale (veterinarian 1 agrees)”.*

Farmers were also concerned with how data would be used and queried whether it would be used for corporate gain by pharmaceutical companies. Farmers discussed the possibility that pharmaceutical companies could become affiliated with the device and ensure that the results of the device point them towards using their products.

### User requirements

For this to be an effective detection tool, participants express a number of key user requirements including reliability of results and key design features.

#### Reliability and trust

Whilst participants expressed a positive reaction to the idea of a diagnostic tool, these sentiments were almost always followed up by remarks of *if* it works, *if* the results are reliable, and *if* it can be trusted. User trust in results is vital to the success of the device, without which, the device will be totally dismissed.

Veterinarian 9: *“You’d want to have confidence in it or else (…), you’d be throwing it away fairly quick”.*

Farmers in particular expressed an apprehension and scepticism in regard to the trust of and reliability of testing, recalling past scenarios where questions surrounded diagnostic testing; for example, cases of false positives in cattle tuberculosis (TB) testing and with human Covid-19 antigen tests. Knowledge of these past failings further cement the need of farmers to have trust in not only the reliability of the testing, but in the *consistency* of accurate testing. It also consolidates the importance of and need for veterinarian involvement and intervention. As veterinarians understand diagnostic testing limitations, they could interpret potentially false negative or false positive test results. Therefore, as one farmer states, it would be more important to get veterinarians convinced that the device works and that they trust it. The importance of getting this level of trust by veterinarians is highlighted in the statement by one veterinarian below.

Veterinarian 7: “*I don’t know how you’d measure that confidence that you have in it. I suppose ultimately will it be ‘oh this is too big a deal to get wrong I’ll just send them to [the] RVL or I’ll send the samples somewhere else’. You’d have to get to that level of trust that you’re [as] confident in it that you are sending it away*”.

#### General design features

Both farmers and veterinarians express similar needs in respect to how this device should be built for use in practice. Farmers in particular state that they would need the tool to be user friendly and straight forward. Both sets of participants indicate that the device should be made robust, rugged, and durable so as that it is not affected by ground conditions. Veterinarians especially stress the importance of durability, stating that there is a large cohort of farmers who you probably would not be able to build a device robust enough for, describing that farmers would have it *“thrown in a bucket in the pit of the parlour”*, or veterinarians would have it *“thrown up on the dash of the jeep”.* If the device is not built to satisfy user needs or to work in the environment in which it will be used, as this farmer puts it, the device will be totally disregarded.

Farmer 15: *“If it’s not built right, durable like and all that and it ends up that it’s kind of going to be a shitty enough device in a years’time, lads will just [say] that’s the end of that (emphasized) as a (…) product really so that’s what could be a problem down the road (…)”.*

## Discussion

This research outlines the current issues with and barriers to animal disease diagnosis on Irish dairy farms. The biggest issue with diagnosis is inadequate testing which has negative associated knock-on effects including treatment delays, increased/unnecessary use of antimicrobials, and detriments to animal health. The research also gives insight into farmer and veterinarians perceptions of the current use of digital technologies for animal health, as well as their initial thoughts on the conception of developing an on-farm diagnostic tool to address aforementioned issues. As well as directly discussing the potential of this diagnostic tool, this research also opens a broader discussion on the implementation of digital technologies for animal disease diagnosis and testing.

### Value of a diagnostic tool to reduce antimicrobial use and to alleviate workload

There is potential for development of a testing tool to alleviate some of the animal health challenges facing the agricultural sector in Ireland. The tool could bring value to the animal health sector by playing an important role in helping Irish farmers and veterinarians comply with and adapt to the rules and associated knock-on effects of newly introduced veterinary medicine regulations by supporting antimicrobial stewardship on farms. There is a requirement of veterinarians to prescribe antimicrobials more responsibly and research has found that a lack of access to rapid diagnostics is a key barrier to appropriate antimicrobial prescribing [12], and that there is a need amongst veterinary practitioners for faster animal health testing [22]. Farmers and veterinarians in this research have highlighted that a rapid diagnostic tool would address this requirement and other diagnostic issues identified in this study, by helping to achieve early diagnosis and enabling users to quickly identify what strain of an illness an animal has. This would facilitate the timely treatment of that illness, leading to more targeted and reduced use of antimicrobials on farms. Similarly, the tool could be used to quickly identify illnesses which do not require an antimicrobial (e.g., viruses), helping to reduce the ‘stop-gap’ use of what turns out to be unneeded antimicrobials. Additionally, it has been highlighted in this study that a testing tool would be useful in aiding farmers in the transition to SDCT practices on farm. These outcomes combined would help Irish farmers and veterinarians in addressing and combating AMR.

This technology can provide more information to farmers which can assist management practices on-farm. The Irish dairy industry operates a seasonal calving system, making springtime one of the busiest times of the year on Irish dairy farms. Managing herd health becomes more of a challenge during this time as a more compact calving means more calves being reared in a shorter window, resulting in challenges such as increased disease pressure on farm. It is expected that calving time may become busier for farmers and veterinarians, as recently published Irish Cattle Breeders Federation (ICBF) figures show the 2022 average six-week calving rate increased to 66% (an improvement of 7% over the past 10 years), with the top 10% of dairy farmers calving 86% of the herd in the first 6 weeks [23]. If these trends continue to increase towards the industry target of 90%, calving time will continue to get busier, meaning managing and maintaining good animal health will become a more important and difficult task for farmers and veterinarians, especially as animals are housed indoors during this time period. Utilising an on-farm testing tool could alleviate disease pressure in these scenarios by enabling users to achieve early diagnosis and intervention, helping to prevent spread of disease to the rest of the herd. This device could also be used by farmers as a general decision-making tool to inform farm management and animal husbandry practices. For example, if the tool identifies widespread respiratory issues on farm, it could indicate that a vaccination programme should be implemented the following year.

### Important considerations for a diagnostic tool

However, it cannot be assumed that the results from this testing device will always be acted upon correctly. Having a rapid testing tool like this has the potential to provide farmers and veterinarians with a greater degree of information and knowledge. Farmers in the current study identified a knowledge gap in their capacity to identify and treat certain animal health conditions and they anticipate that digital technology can close that gap. However, although this expectation exists, it does not necessarily mean that it will be met. Precision livestock farming technologies are developed with the premise that users will have access to more and better information, assisting with decision making [24]. Whilst digital technologies can offer potentially valuable information, it does not necessarily mean that users will completely understand or utilise it, highlighting the important but often overlooked difference between adoption and use. Whilst these tools are monitoring animals and generating information, it is important that users are monitoring this information, and acting on it appropriately.

Additionally, when using technology, there is a risk of misinformation and incorrect use of information, and concerns regarding the use of information. While the main aim and premise for developing this testing tool may be to reduce overall antimicrobial use on farms, the current study found that there is a risk that the tool could lead to an over-prescribing of antimicrobials; if the testing tool is inaccurate, or if the testing results are acted upon incorrectly by users i.e., the prophylactic use of antimicrobials. This would result in a possible mismatch between technology intention and outcome and re-emphasises the importance of accuracy of animal health diagnostic technologies. Participants in this research recognised that farmers and veterinarians could not be totally dependent on the results of a technology like this to make a diagnosis, therefore, a testing tool like this should be used as an aid or a support tool in a diagnostic work up. Using the tool in this way would increase trust of the device and mitigate possible misinterpretation of test results. This study also identifies concerns regarding how information and data from technologies will be used, and whether it will be used to benefit larger corporations such as pharmaceutical companies instead of, or at the expense of, the user (i.e., the farmer or veterinarian); an issue that has been of growing concern in the digital agriculture space [17]. It is also worth noting that farmers in this study highlighted that although they can use this technology as a tool to identify animal health issues on farm, they still want to consult their veterinarian for reassurance and advice on what steps to take next. This indicates that although they are gaining information from the device, farmers still value and want input from their veterinarian, consolidating the importance of the farmer-veterinarian relationship.

### Development of digital technologies for veterinarian use

The current study highlights the importance of employing user-centred design in PLF as this approach to technology development can help to identify and alleviate such unintended consequences at an early stage. To overcome these potentially unintended negative consequences, Responsible Research Innovation (RRI) principles should be applied to future technology design. Underpinning the concepts of RRI (anticipation, inclusion, reflexivity, responsiveness) to technology design will ensure that future technologies will be conscious of and responsive to the concerns, needs, and expectations of key end users and society as a whole [16, 25]. This study has demonstrated the value of some of these principles as engagement with future end users (inclusion) has successfully identified needs, concerns, and potential negative impacts (anticipation) that an on-farm testing tool could have. We suggest that if this tool is to be developed for commercial use, technology developers and engineers should be responsive to the needs and concerns of farmers and veterinarians highlighted in this study. As well as this, technology developers should continue to engage with end users in the tools future development, to continue to anticipate and be responsive to other yet unidentified problems and needs.

The testing technology has been identified in the current study to act as a decision support tool which could aid and alleviate some of the heavy workload and frustrations experienced by veterinary practitioners. Retention and recruitment of veterinary practitioners is a real area of concern in Ireland [26]. Veterinarian shortages are predicted to worsen in years to come with Ryan and co authors [26] finding that just over half of Irish veterinarians who responded to their survey (*n = 370*) are considering leaving their role within the next 2 years, citing issues of work/life balance as the top reason for them wanting to leave. The current research has highlighted some of the implications that these veterinarian shortages are having, including the impact it can have on veterinarian farm visits and subsequent diagnosis. Digital technologies can help address these issues. Within the agricultural sector, there is a focus on the narrative of digital technology development to address *farmer* labour availability concerns; to make the agricultural industry a more attractive occupation for new *farmer* entrants; promising an improved work life balance for *farmers*; and offering a means for *farmers* to manage animal health on farms [17]. However, there is less reported in the digital agriculture literature on the development of precision livestock farming tools which are aimed at veterinarians. We argue that similar sentiments and efforts that are being applied to develop technology to help farmers along should be applied to the veterinary sector.

This research has made an initial effort at developing digital tools for veterinarian use. Engaging with end users to situate the concept of this testing tool in context and working with them to imagine how this concept would be employed in a larger setting has been useful in establishing potential for and a need for the development of a rapid on-farm animal health testing tool. It has also been valuable in identifying initial user needs and primary requirements of farmers and veterinarians for a testing tool like this. Future research would benefit from building on these needs and requirements, and we suggest that co-design approaches should be used to further develop the concept of this tool. Such approaches include Design Thinking, an approach long established in other fields, but which is a relatively new but growing application of design in the agricultural sector. Design Thinking is an approach with represents a shift in attitude from designing *for* users to one of designing *with* users [27]. During the five-stage process, researchers, technology designers, and end users collaborate; with end users being placed at the core of the design and development process. Reflexive and iterative engagement with end users ensures that user needs and preferences are highlighted, and that products and services are specifically designed to address those values and needs. Design Thinking tools have been used recently in an agricultural context to design a geo-tagged photo app [13, 28], in the development of PLF strategies for arid grazing regions [29], and in redesigning dairy systems in New Zealand [30]. The authors therefore suggest that the use of similar approaches should be applied to the future design and development of animal health diagnostic tools.

### Study limitations

We do not claim that the study findings represent the full range of experiences and viewpoints of all farm animal veterinarians and dairy farmers in the Republic of Ireland. As the main recruitment strategy for farmers was via agricultural advisors, we can assume that the farmers at the focus groups were actively involved in information seeking behaviour. The participating farm animal veterinarians actively put themselves forward for participation in this study, therefore can be considered to be actively involved in research and innovation development. Responses therefore may not reflect the opinions of the majority of Irish farmer and farm veterinarian populations.

## Conclusion

The threat of AMR coupled with newly introduced veterinary medicine regulations are placing Irish farmers and veterinarians under increasing pressure to reduce antimicrobial usage at farm level. Digital technologies can support farmers and veterinarians in reducing antimicrobial use on farm and this study has been successful in exploring the potential of a rapid on-farm animal health diagnostic tool to achieve this. Applying a user centred design approach to the development of this tool has been successful in identifying initial user needs and requirements for a testing tool. However, as well as identifying benefits, engagement with future end users has found that improper development could have unintended negative impacts such as misdiagnosis, increased antimicrobial use, and issues of adoption. Therefore, in order to mitigate future negative impacts, and to ensure the successful development and adoption of an on-farm testing tool, co-design approaches such as Design thinking should be applied to its design and development.

### Supplementary Information

**Supplementary Material 1.**

## Data Availability

The dataset used in the current study are available from the corresponding author on reasonable request.

## Abbreviations

AMR: Antimicrobial Resistance
WHO: World Health Organisation
EU: European Union
BDCT: Blanket Dry Cow Therapy
SDCT: Selective Dry Cow Therapy
RVL: Regional Veterinary Lab
PLF: Precision Livestock Farming
TB: Tuberculosis
ICBF: Irish Cattle Breeding Federation
RRI: Responsible Research Innovation

## Footnotes

1 On-farm tests available to farmers include the snap test (detect antimicrobial residue in milk), the California Mastitis Test (CMT) (to indicate high SCC quarters i.e., subclinical mastitis), the rainbow calf scour test (to detect rotavirus, coronavirus, e-coli, or crypto scours) and colostrum quality testing.

2 Additionally, vets can test faecal samples for worms and fluke, and milk samples for basic gram positive or gram-negative cultures. Ketotic testing can be used to diagnose sub-clinical ketosis, and serum total proteins can be tested in calves to assess passive transfer.

3 A Science Foundation Ireland and Department of Agriculture Food and the Marine (DAFM) funded research centre based in the South of Ireland

4 A leading European Research Centre in integrated ICT hardware and systems based in the South of Ireland
