# Supplementary material for: Development of an animal health testing tool to reduce antimicrobial use on farms: perceptions, implications, and needs of Irish dairy farmers and farm veterinarians
Source: Ir Vet J. 2024 Jun 21;77:12. doi: 10.1186/s13620-024-00268-x (PMC11191197; doi:10.1186/s13620-024-00268-x)
Supplement: Supplementary file 1 — Supplementary Material 1. [file 13620_2024_268_MOESM1_ESM.docx]

**Appendix A**

*Part 1. To identify key animal health issues on Irish dairy farms including issues with their detection and diagnosis.*

1. What are some of the key issues / concerns you have in regard to animal health & welfare on your farm?
2. What problems or barriers do you face in terms of detecting and diagnosing animal health & welfare problems/concerns on your farm?

*Part 2. To explore the potential of a digital tool to address detection issues. (The concept of the digital tool was explained to participants).*

1. Would you use a tool like this?
2. Do you think a tool like this would be useful?
3. Where on the farm would a tool like this be of most use?
4. What do you think a tool like this should look like?
5. Would you have any concerns regarding using a tool like this? Anything that would prevent you from using it?
